# Evaluation of Fertilizer Potential of Different K Compounds Prepared Utilizing Sea Bittern as Feed Stock

**DOI:** 10.3389/fpls.2017.01541

**Published:** 2017-09-07

**Authors:** Khanjan Trivedi, Denish Kubavat, Krishna K. Ghara, Ranjeet Kumar, Hardik Trivedi, K. G. Vijay Anand, Pratyush Maiti, Arup Ghosh

**Affiliations:** ^1^Academy of Scientific and Innovative Research, CSIR-Central Salt and Marine Chemicals Research Institute Bhavnagar, India; ^2^Plant Omics Division, CSIR-Central Salt and Marine Chemicals Research Institute Bhavnagar, India; ^3^Process Design and Engineering Division, CSIR-Central Salt and Marine Chemicals Research Institute Bhavnagar, India

**Keywords:** sea bittern, schoenite, maize, potash, fertilizer, soil quality

## Abstract

**Aim:** Many countries import potassic fertilizers due to dearth of K-mineral deposits. Therefore processes to obtain K-nutrient sources from sea bittern were developed by our Institute. The present investigation evaluated the fertilizer potential of three different sea bittern-derived (SBD) potassium forms developed viz., potassium schoenite, potassium nitrate and potassium ammonium sulfate on maize productivity in two cropping seasons.

**Methods:** The pot and field experiments consisted of four treatments, wherein the three K forms were applied at the recommended rate of 40 kg K_2_O ha^−1^ and were compared with commercially used sulfate of potash. The effect of these fertilizers on different parameters of plant and soil were evaluated.

**Results:** The application of SBD-potassic fertilizers led to enhancement in growth, productivity and quality of maize which related well with higher photosynthesis, nutrient uptake and soil quality parameters. On an average all the three forms of sea bittern-derived potash enhanced yield of maize over control by 22.3 and 23.8%, respectively, in pot and field trials. The best performance was under SBD-KNO_3_, which also recorded the highest benefit: cost ratio of 1.76.

**Conclusion:** The K-fertilizers derived from sea-bittern—a waste product of salt industry—can thus be economically used to improve crop production sustainably.

## Introduction

Potassium plays an important role in plant growth, development, defense, immunity, signaling, and transport processes (Beringer and Troldenier, 1980) and is therefore crucial for obtaining good yield and quality of crop plants (Zörb et al., 2014). It also helps plants to adapt to various environmental stresses such as drought and salinity (Anschütz et al., 2014). Despite being one of the most abundant elements in the earth's crust (2.3%), potassium is not easily available to the plants at once from the soil as most of it (90–98%) is present in chemically bound forms which is either unavailable or slowly available (Römheld and Kirkby, 2010). Thus soil reserve has to be supplemented with potassic fertilizers for crop production. Since 1980, about 25% increase has been found in the use of potassic fertilizers (Zörb et al., 2014). Cereals have the maximum share (FAO, 2011) for potassium use. Predominantly, potassium nutrient is applied to the crops through muriate of potash (KCl), which accounts for 96% of the world's potash capacity (Kinekar, 2011). However, other fertilizer forms such as sulfate of potash (SOP), potassium ammonium sulfate (PAS), potassium nitrate (KNO_3_) are also used depending upon the availability and cost. However, sufficient potassic mineral deposits are not present in many countries for producing potassic fertilizers; for example, India imports 100% of its potassic requirement from Canada, Belarus, CIS, Jordan, Israel, UK and Germany (Kinekar, 2011) which for 2013–14 was 1.926 million tonnes. China has to import about 55% of its potash requirement. Other Asian countries (except Middle East, India and China) also are fully dependent on import to fulfill their potassic demand. Similarly, the bulk of Brazilian potash is also shipped from countries like Belarus, Russia, Canada, Israel etc. Even though North American domestic production is high, it still has to rely on imports to meet some of its requirement (TFI World Fertilizer Conference, 2015). Thus it is pertinent to devise technologies to produce potassic fertilizers from other potash rich sources. In the pursuit of manufacturing potassic fertilizers through other feed stocks, we developed few processes of making potassium containing compounds using potash rich sea bittern which may have fertilizer potential (Ghosh et al., 2011, WO 2010109492 A8; Vohra et al., 2004, US 6776972 B2). Bittern is the mother liquor left after solar salt production from brines and/or seawater which is rich in potassium. The bittern also contains other elements like magnesium, chlorides, sulfates, bromides, iodides. For every 1 ton of common salt produced, at least 1.0 m^3^ of bittern is left behind (personal communication with Dr. Arvind Kumar). Given the fact that about a quarter billion tons of salt is produced in the world, of which about 40% is produced by solar evaporation technique (Sedivy, 2012), the amount of bittern generated offers tremendous scope to produce potassic fertilizers. The present investigation sought to evaluate the fertilizer potential of three different forms of sea bittern-derived (SBD) potassium *viz*., K schoenite (K_2_SO_4_.MgSO_4_), KNO_3_ and PAS for crop production. Detailed study was done to assess the efficacy of these K-compounds on plant and soil in a pot study followed by field trial using maize as a test crop so as to establish their fertilizer potential in crop production.

## Materials and methods

### Production of potassic compounds

SBD-KNO_3_ was produced by selective precipitation method using tartaric acid as precipitant from sea bittern (Ghara et al., 2014; Maiti et al., 2014) Tartaric acid was treated with bittern upon which selectively, potassium was precipitated as potassium bitartrate. Potassium bitartrate was decomposed by Mg(OH)_2_ and HNO_3_ to yield magnesium tartrate that precipitated as a solid and KNO_3_ as liquor. Solid SBD-KNO_3_ was obtained through cooling crystallization of KNO_3_ liquor. SBD-Potassium ammonium sulfate was also produced by tartaric acid selective precipitation method from bittern. When potassium bitartrate was treated with MgSO_4_ and NH_4_OH, magnesium tartrate that precipitated as a solid and SBD-PAS in solution were produced. The SBD-PAS salt was then crystallized from solution through forced evaporation. SBD-K schoenite was produced through the decomposition of kainite mixed salt (KCl·MgSO_4_·3H_2_O) which was obtained from sea bittern (Ghosh et al., 2005; Dave and Ghosh, 2006; US 20050220698 A1). Elemental composition and salinity index (SI) of these compounds are presented in Table 1. SI was determined according to Jackson (1958) by following equation:

$$\text{SI}=\frac{\text{Specific}\;\text{conductance}\;\text{of}\;\text{solution}\;\text{when}\;\text{1}\;\text{g}\;\text{of}\;\text{fertilizer}\;\text{is}\;\text{suspended}\;\text{in}\;\text{1}\;\text{liter}\;\text{of}\;\text{water}\;}{\text{Specific}\;\text{conductance}\;\text{of}\;0.1\%\;\text{sodium}\;\text{nitrate}\;\text{solution}}\times 100$$

**Table 1:** Elemental composition and salinity index of different K compounds used in the experiment.

**Different K compounds**	**Parameters (% w/w)**								**Salinity index**
	**K**	**Mg**	**Ca**	**${\text{NH}}_{4}^{+}$**	**${\text{SO}}_{4}^{2-}$**	**Na**	**Cl^−^**	**${\text{NO}}_{3}^{-}$**	
Potassium nitrate	38.9	^*^BDL	BDL	BDL	BDL	BDL	BDL	60.8	95.1
Potassium ammonium sulfate	18.6	3.5	0.28	6.02	60.4	BDL	0.88	BDL	102.0
Potassium schoenite	24.4	4.3	0.74	BDL	47.8	1.44	3.52	BDL	83.3
Commercial grade SOP	50.0	BDL	BDL	BDL	51.0	0.88	0.89	BDL	112.7

* *BDL, below detection limit*.

### Study area and experimental design

The experiment was carried out during two cropping seasons at varied locations during 2014–15. Both the trial locations are located 35 km apart. The region receives about 555 mm of rainfall annually and has a mean maximum and minimum temperature of 35 and 19°C, respectively.

#### Pot experiment

The pot experiment using maize (sweet corn, variety Sugar 75, Syngenta) as test crop was conducted at the net house facility (21°44′57.6″N latitude, 72°08′39.3″E longitude) of CSMCRI, Bhavnagar district, Gujarat, India during *Kharif* season (July to October 2014). The treatments comprised of three different potassic compounds produced from sea bittern, viz., SBD-K schoenite (T2), SBD-KNO_3_ (T3) and SBD-PAS (T4) which were compared to commercial grade sulfate of potash (SOP) currently available in the market (control; T1). Each treatment was replicated five times and laid out in completely randomized design (CRD). All the pots were filled with 32 kg of soil to which chemical fertilizers at the recommended rate of 120:60 kg ha^−1^ of N: P_2_O_5_ were applied uniformly to all the treatments through urea and single super phosphate (SSP), respectively. Potassium as K_2_O was applied at 40 kg ha^−1^ to all the treatments through different fertilizers depending upon their potassium content. In the treatments receiving potassium nitrate and potassium ammonium sulfate, the balance amount of nitrogen was supplied through urea. Two liters of water was applied to each pot every third day. The soil of pot experiment was sandy loam in texture, having initial pH 7.88 with 0.22 dS m^−1^ electrical conductivity. Available N, P, and K were 49, 6.70, and 74 mg kg^−1^, respectively. Organic carbon was 1.71%. Four seeds were sown in each pot, which after successful germination was thinned to single plant per pot.

#### Field experiment

A trial comprising the same treatments was repeated in field in bigger sized plots at Neswad farm (Altitude-314 ft, N 21°30′494″ E 072°02′185″) of Bhavnagar district, Gujarat, India during *Rabi* season (November 2014 to February 2015). The soil was Entisol, classified according to the USDA soil taxonomy as isohyperthermic, mixed-loamy kaolinitic, lithic ustorthents. It was also sandy loam in texture, having initial pH 6.97 and 0.14 dS m^−1^ electrical conductivity. Available N, P and K were 74, 1.39 and 117 mg kg^−1^, respectively. Ca and Mg were 3.62 and 2.22 meq 100g^−1^, while S was 99 mg kg^−1^. Organic carbon and organic matter were 0.41 and 0.71%, respectively. Here each treatment was replicated thrice and was laid out in randomized block design (RBD) with gross plot size of 4 × 3 m and the measurements were taken from net plot size of 3 × 2 m. Chemical fertilizers at the recommended rate of 120:60:40 kg ha^−1^ of N: P_2_O_5_: K_2_O were applied uniformly to all the treatments. Nitrogen was applied in three doses during the entire life cycle of maize. One fourth of nitrogen was applied as basal application before sowing while remaining quantities of nitrogen was divided into two equal doses and applied as top dressing at the knee-high stage and tasselling stage, respectively. The exact amount of applied fertilizers in g (gross plot)^−1^ were as follows: (1) 96 g Commercial-SOP, (2) 205.6 g SBD-K schoenite, (3) 102.4 g SBD-KNO_3_, (4) 156.3 g SBD-PAS, (5) 450 g SSP, (6) 313.0 g urea (in T1 and T2), (7) 294.5 g urea (in T3), and (8) 294.4 g urea (in T4). The spacing between two rows was maintained 60 cm and plant-to-plant distance was 20 cm, with a plant population of 83,333 ha^−1^. Data on agronomic parameters was collected from plants within the net plot area from each plot for further analysis. Five furrow irrigations at the rate of 50 mm were applied during the whole period of the experiment. Meteorological data for the field experimental is presented in the Supplementary Table.

### Growth, yield and photosynthetic parameters

The growth, yield and other yield attributes were recorded at harvest. Plant height was measured as the distance from soil to the last leaf collar, while stem diameter was measured at the base 3 cm above the soil surface. Leaf area was measured with leaf area meter (CI-202, CID Inc., USA). Fresh, sundried as well as oven dried weight of the plant parts (grain, leaf, stem and root) were recorded after harvest. Roots were carefully removed from soil and shaken by hand, sieved to remove the sand particles. Root fibers were then cleaned by moist paper towel until complete removal of the soil particles from root tissue. Fresh root weight was recorded at this point of time and after which root volume was measured by water displacement method. To measure root volume, samples were submerged into 1 liter graduated cylinder filled with 500 ml water. Water volumes in the graduated cylinder were recorded before as well as after submerging and root volume was calculated as follows: Root volume = volume of the water after submerging the roots into the cylinder—volume of the water before submerging the roots (Pang et al., 2011). Measurements on cob were done just after harvest of fresh cobs. The grains were separated from the cobs after sun-drying and were expressed as per plant and hectare basis in pot and field trial, respectively. In the field trial, yield data were collected from the net plot area of 3 × 2 m by manually harvesting the cobs. Further five plants were selected randomly from each plot and were used for recording data of plant height, fresh cob attributes (weight, length as well as for other measurements), root weight and other yield parameters like grain fill length. Photosynthetic rate (PR), transpiration rate (TR) and other gas exchange parameters were measured from the flag leaf of each replicates using infrared gas analyzer system (IRGA; Model Li-6400XT, LI-COR, USA) at the photosynthetic photon flux density (PPFD) of 1,000 μmol m^−2^ s^−1^. CO_2_ in the leaf chamber was maintained at a fixed concentration by allowing air to continuously pass through leaf chamber from an open end, it being an open photosynthesis gas exchange system. Water use efficiency (WUE) defined as the net productivity per-unit water transpired, was calculated as the ratio of photosynthesis and transpiration (PR/TR). Initial values of the minimum (F_o_) and maximum (F_m_) fluorescence yield were recorded in the dark state of leafs (before dawn). Remaining chlorophyll fluorescence parameters. *viz*., F_v_/F_m_, SC (stomatal conductance), qP (photochemical quenching), ΦPSII (quantum yield of PS II electron transport), NPQ (non-photochemical fluorescence quenching), ETR (photosynthetic electron transport rate), ΦCO_2_ (apparent quantum yield of CO_2_ assimilation), C_i_ (intercellular CO_2_ concentration), C_a_ (ambient CO_2_ concentration) and C_i_/C_a_ ratio were calculated as reported by Maxwell and Johnson (2000). Chlorophyll index (CI) was measured using Chlorophyll content meter (Model CCM-200, Opti-Sciences Inc., USA). Three leaves per plant and total five plants in each treatment were used to measure chlorophyll content. The three leaves were flag leaf, one above and one below the flag leaf. Water content of the plant parts was estimated by gravimetric method after oven drying the material at 80°C till constant weight and expressed as ml per plant part.

### Plant and soil analysis

All leaves, stem, root and grains of individual plants were collected, homogeneously crushed and powdered for nutrient content analysis. N, P and K were determined by digesting the plant samples with sulfuric acid-selenium-salicylic acid mixture as described by Novozamsky et al. (1983) followed by estimation using continuous flow analyzer (San++ SKALAR) where N was estimated by colorimetric Berthelot reaction (Krom, 1980; Searle, 1984), P was estimated by formation of a blue-colored phosphomolybdenum complex by reduction with ascorbic acid and K was estimated by flame photometric method described in Plant Analysis Procedures (Ed. Temminghoff and Houba, 2004). Ca and Mg were determined by digesting the powdered plant samples in di-acid mixture (nitric acid and perchloric acid) as described by Miller (1998) followed by estimation through inductively coupled plasma optical emission spectrometry (ICP-OES, Optima 2000, PerkinElmer).

For soil analysis, immediately after harvest, core samples 5 cm diameter from 0 to 20 cm depth were drawn randomly from 3 regions, pooled to get a composite mixture per replicate. Samples were further sieved (< 2 mm) and stored at 4°C prior to biochemical analysis. All the enzymatic and microbial parameters were determined in pot experiment and analysis was carried out within 2 weeks of sample collection, while the other physico-chemical analyses were completed within 8 weeks. pH and electric conductivity (EC) were determined in 1:2.5 slurry of soil: water. Water holding capacity and moisture content were determined by gravimetric method (drying the soil at 105°C until the constant weight was achieved). Organic carbon, total-, available- nitrogen and available P were determined by Walkley and Black procedure as described in Nelson and Sommers (1982), modified Kjeldahl method using salicylic acid by Wilke (2005) and Olsen's method (Olsen and Sommers, 1982), respectively. Na and K were extracted by neutral normal ammonium acetate (Hanway and Heidel, 1952) and determined by using flame photometer. Ca and Mg were determined by EDTA titrimetric method as described by Estefan et al. (2013). S was extracted with 0.15% CaCl_2_ (Williams and Steinbergs, 1959) and estimated by turbidimetric method as described by Estefan et al. (2013). Organic matter was calculated from organic carbon using multiplication factor of 1.724 (Howard, 1965). Aryl sulphatase, acid and alkaline phosphomonoesterase and glucosidase activities in the field moist soil was assayed according to Tabatabai (1982) using 4-nitrophenol as standard. FDA activity was measured according to Schnürer and Rosswall (1982).

### Statistical analysis

Statistical analyses were carried out using MSTAT C software (Michigan State University, East Lansing, MI) suitably employing completely randomized design (CRD) and randomized block design (RBD) procedures for pot and field experimental data, respectively. *Post hoc* comparison of means was carried out using Tukey's HSD at a significance level of *p* < 0.05.

### Economic analysis

The general cost of cultivation comprised of all the costs incurred in maize cultivation sans potassic fertilizer and nitrogenous fertilizers. Notably, N through urea was applied to the SBD-KNO_3_ and SBD-PAS treatments after discounting the N amounts that gets inadvertently applied through these fertilizer forms. Thus, the incremental costs included the varying cost of N and K fertilizers for the purpose of economic calculation and together along with general cost was taken as the total cost of cultivation. The cost of commercially available NPK fertilizers, viz., urea, single superphosphate and sulfate of potash used in the study were taken as 6.27, 6.5, and 40 INR kg^−1^, while the cost of indigenous sea bittern derived K-fertilizers, viz., K-schoenite, KNO_3_ and PAS was deduced to be 10, 40, and 16 INR Kg^−1^ on the basis of process cost data generated at pilot plant in the Institute. The total gross returns included the cumulative price realized on account of maize grains, stone and stalks based on the prevailing market rates which were 13,650, 1,000, 1,000 INR Kg^−1^, respectively. The net return represented the difference between the total gross returns and the total cost incurred per hectare. The benefit: cost ratio (B : C ratio) was calculated as the ratio of total gross return and total cost of cultivation (Gahoonia et al., 2005).

## Results

The effects of different bittern-derived potassic nutrient treatments on maize crop were examined in terms of crop growth attributes, photosynthetic efficiency, yield attributes, yield, nutrient uptake, soil physico-chemical and biochemical parameters and through economic analysis (Tables 2–**6**).

**Table 2:** Growth, yield and yield attributes of maize as affected by different sea bittern-derived potassic nutrient treatments in pot experiment.

**Parameters**				**Treatments**			
				**Commercial ^1^SOP (control)**	**^2^SBD-K schoenite**	**SBD-KNO_3_**	**SBD-^3^PAS**
**GROWTH ATTRIBUTES**							
Plant height at harvest (cm)				178^b^	211^a^	207^a^	209^a^
Basal stem diameter at harvest (mm)				20.54^c^	22.45^ab^	24.09^a^	21.71^bc^
Dry matter accumulation (g plant^−1^)	Grain			40.5^c^	48.9^ab^	54.8^a^	47.4^b^
	Leaf			49.2^b^	59.8^ab^	61.8^a^	55.3^ab^
	Stem			30.5^b^	50.1^a^	57.0^a^	53.1^a^
	Root			29.4^b^	40.0^a^	36.6^a^	28.9^b^
	Total			149.6^b^	198.8^a^	210.1^a^	184.7^a^
Moisture content of various plant parts (ml)	Leaf			76.50^c^	90.40^bc^	116.34^a^	97.48^b^
	Stem			138^b^	190^a^	176^a^	168^a^
	Root			20.60^b^	11.48^b^	51.12^a^	50.74^a^
Total leaf area (m^2^ plant^−1^)				0.35^a^	0.40^a^	0.35^a^	0.38^a^
Root volume (cc plant^−1^)				110^c^	110^c^	210^a^	152^b^
**YIELD AND YIELD ATTRIBUTES**							
Fresh cob parameters	With leaf cover	Cob weight (g)		225^c^	274^b^	302^a^	279^b^
		Cob length (cm)		23.06^b^	25.56^a^	26.10^a^	26.64^a^
	Without leaf cover	Grain fill length (cm)		15.50^b^	17.48^ab^	18.30^a^	15.68^b^
		Diameter (mm)	Lower	46.16^a^	46.40^a^	47.60^a^	46.18^a^
			Middle	43.71^a^	43.74^a^	43.53^a^	42.76^a^
			Top	40.20^a^	41.77^a^	40.60^a^	41.20^a^
Sundried cob parameters	No. of seed rows per cob			14.6^a^	14.8^a^	14.4^a^	15.0^a^
	No. of seeds			528^b^	587^a^	559^ab^	550^ab^
	Total grain weight (g)			43.7^c^	51.0^b^	57.6^a^	51.7^b^
	100 seed weight (g)			7.69^c^	8.32^*bc*^	9.79^a^	8.62^b^
Grain carbohydrate	Yield (g plant^−1^)			30.24^c^	38.63^ab^	43.13^a^	37.09^b^
	Content (%)			74.64^b^	79.00^a^	78.78^a^	78.22^a^
Grain protein	Yield (g plant^−1^)			3.01^a^	3.03^a^	2.81^a^	2.55^a^
	Content (%)			7.42^a^	6.21^a^	5.10^a^	5.37^a^

*Values represented are mean of 5 replicates. Values followed by different alphabets in the rows are significantly different at p < 0.05 using Tukey's HSD*.

1 SOP, sulfate of potash;

2 SBD, Sea bittern-derived;

3 *PAS, Potassium ammonium sulfate*.

### Growth attributes

Growth attributes of maize plant were altered significantly due to different K fertilizers (Tables 2, 3). In pot experiment, compared to control (commercial sulfate of potash) plant height and diameter at harvest were significantly increased by all the three bittern-derived potassic nutrient treatments, viz., SBD-K schoenite, SBD-KNO_3_ and SBD-PAS. Maximum plant height was observed in SBD-K schoenite treated plants which was at par to the other two bittern-derived potash treatments. Maximum basal stem diameter was recorded in SBD-KNO_3_ treated plants which was closely followed by SBD-K schoenite and both these treatments were superior to SBD-PAS and control. Dry matter accumulation in different plant parts (grain, leaf, stem and root) has also been presented in Table 2. The highest total plant biomass was found in SBD-KNO_3_, which was however at par with all other bittern-derived potassic nutrients, all of which were superior to control, connoting improved stover production. Other than in leaf, dry matter accumulation in other plant parts was lower in control as compared to that in SBD-K schoenite and SBD-KNO_3_ treated plants. SBD-PAS and control treatments were however at par to each other with respect to dry matter accumulation in roots and leaves. SBD-KNO_3_ treated plants had the highest degree of hydration in its vegetative biomass and contained about 45% higher water content (dry basis, w/w) compared to control. This treatment recorded the highest root volume compared to all other treatments and also recorded the highest water content in leaves. All the three bittern-derived K treatments were at par with each other with respect to water content in stem but were superior to control. There was no change in leaf area per plant due to any of the bittern-derived nutrient treatment (Table 2). Results of growth yield and yield attributes of field trial has also been shown in Table 3. In the field trial, the highest plant height was found in SBD-KNO_3_ treated plants which was at par with SBD-PAS and SBD-K schoenite applied treatments. However, SBD-K schoenite was found at par with control that recorded the lowest value for this parameter.

**Table 3:** Growth, yield and yield attributes of maize as affected by different sea bittern-derived potassic nutrient treatments in field trial.

**Parameters**		**Commercial ^1^SOP (control)**	**^2^SBD-K schoenite**	**SBD-KNO_3_**	**SBD-^3^PAS**
Plant height at harvest (cm)		167.1^b^	176.8^ab^	183.5^a^	180.7^a^
Cob weight with leaf cover (g)		343.1^b^	406.1^ab^	411.3^a^	406.7^ab^
Cob length without leaf cover (cm)		17.25^b^	19.40^a^	19.10^a^	19.16^a^
Cob diameter without leaf cover (mm)	Bottom	48.03^a^	51.86^a^	52.09^a^	51.99^a^
	Middle	46.90^a^	51.31^a^	51.68^a^	50.31^a^
	Top	43.62^a^	50.05^a^	53.24^a^	47.88^a^
Root weight (g)		34.99^b^	82.87^a^	58.97^ab^	67.90^a^
Grain fill length (cm)		15.37^b^	18.73^a^	17.12^ab^	17.61^ab^
100 seed weight (g)		8.28^c^	8.68^c^	10.30^a^	9.41^b^
Yield (t ha^−1^)		4.08^b^	5.02^a^	5.12^a^	5.01^a^

*Values represented are mean of 3 replicates. Values followed by different alphabets in the rows are significantly different at p < 0.05 using Tukey's HSD*.

1 SOP, Sulfate of potash;

2 SBD, Sea bittern-derived;

3 *PAS, Potassium ammonium sulfate*.

### Yield attributes

The different bittern-derived nutrient treatments also significantly altered the yield parameters of maize plants in both the pot and field experiments (Tables 2, 3). In the pot experiment (Table 2), the green cob length and weight (inclusive of cob green leaf cover) were significantly higher in all the bittern-derived K treatments compared to that in control. The highest green cob weight was obtained under potassium nitrate treatment and this treatment was superior to all others, while in case of green cob length, the SBD-PAS treatment recorded maximum length, but was at par with all other bittern-derived K treatments. The grain fill length on the cob differed due to the different treatments and was the maximum under SBD-KNO_3_ treatment, which was at par with SBD-K schoenite. There was no change in cob diameter (without cob green leaf cover) and number of seed rows per cob due to the treatments. SBD-K schoenite was the only treatment that differed significantly in the number of grains formed per cob, compared to control. 100 seed weight was maximum in SBD-KNO_3_ treated plants, which was 27% higher over control. Test weight in SBD-PAS was significantly higher by 12% over control, while SBD-schoenite was at par for this parameter. Total grain weight was significantly higher in all the treatments compare to control. SBD-KNO_3_ gave the maximum grain weight (57.6g), which was 32% higher than commercial grade SOP. SBD-K schoenite and -PAS were at par to each other with respect to grain yield but were significantly higher than control (17 and 18%, respectively). Significantly the highest carbohydrate content, compared to control, was found in grains of SBD-K schoenite treated plants which was, however, at par with SBD-KNO_3_ and -PAS, while maximum carbohydrate yield was found in SBD-KNO_3_ treated plants, which was at par with SBD-K schoenite but significantly higher than SBD-PAS followed by control, which recorded the lowest. No change in protein content as well as protein yield was apparent due to any of the bittern-derive K treatments.

Similarly in field trial (Table 3), significantly highest green cob weight was also observed in SBD-KNO_3_ treated plants, while all others were at par to each other. Notably, length of cob (without its cob leaf cover) was significantly higher in all the bittern-derived potassic treatments compared to that in control. Similar to the results obtained in pot experiment, no change was found in cob diameter. Root weight was found higher in all bittern-derived potassic treatments compared to that in control. Cob fill length was found maximum in SBD-K schoenite treated plants compared to all others which were, however, at par among themselves. 100 seed weight was significantly highest in SBD-KNO_3_ treated plants, followed by SBD-PAS and SBD-K schoenite. SBD-K schoenite was found at par with control with respect to 100 seed weight. This was more or less similar to that in pot experiment. In comparison to control, yield was found significantly highest in SBD-KNO_3_ treatment, which was however, at par to other two bittern-derived potash treatments.

### Gas exchange parameters

Photosynthetic rate and other gas exchange parameters were also significantly affected by different forms of bittern-derived potash (Table 4). Photosynthesis rate was found significantly highest in SBD-PAS treated plants, followed by SBD-K schoenite and SBD-KNO_3_, both of which were at par to each other. When compared to control, chlorophyll index (CI) was significantly higher in all bittern-derived potassic treatments, while they all were at par to each other. The quantum yield of PS II electron transport (ϕPS2), F_v_/F_m_ ratio, apparent quantum yield of CO_2_ assimilation (ϕCO_2_), electron transport rate (ETR) and water use efficiency (WUE) were significantly enhanced in all the treatments when compared to control. Transpiration rate was the highest in control, being comparable to that in SBD-KNO_3_ and SBD-PAS, while it was significantly lowest in SBD-K schoenite treated plants. Intracellular CO_2_ concentration (Ci) and Ci/Ca ratio was also found significantly higher in different K fertilizers compared to that in control.

**Table 4:** Chlorophyll Index and gas exchange parameters of maize as affected by different sea bittern-derived potassic nutrient treatments in pot experiment.

**Parameters**	**Commercial ^14^SOP (control)**	**^15^SBD-K schoenite**	**SBD-KNO_3_**	**SBD-^16^PAS**
^1^CI	38.21^b^	51.75^a^	55.30^a^	50.10^a^
^2^PR	16.67^c^	22.73^b^	22.57^b^	28.49^a^
Fv/Fm ratio	0.72^c^	0.81^b^	0.85^a^	0.85^a^
^3^SC	0.08^c^	0.14^ab^	0.19^a^	0.12^bc^
^4^C_*i*_ (μmol CO_2_ m^−2^ s^−1^)	129.61^b^	181.20^a^	210.90^a^	186.16^a^
^5^ϕPS2	0.15^b^	0.23^a^	0.26^a^	0.25^a^
^6^ϕCO_2_ (μmol CO_2_ m^−2^ s^−1^)	0.02^b^	0.03^a^	0.03^a^	0.03^a^
^7^qP	0.31^b^	0.47^ab^	0.57^a^	0.55^a^
^8^NPQ	0.80^a^	0.88^a^	1.18^a^	1.09^a^
^9^ETR	66.37^b^	102.81^a^	113.40^a^	108.98^a^
^10^TR (mmol H_2_O m^−2^ s^−1^)	6.91^a^	4.95^b^	5.36^ab^	5.60^ab^
^11^WUE (μmol CO_2_/mmol H_2_O)	2.44^b^	4.62^a^	4.27^a^	5.31^a^
^12^CLT (°C)	40.96^a^	40.77^a^	40.89^a^	41.04^a^
^13^C_*i*_/C_*a*_	0.26^b^	0.38^a^	0.47^a^	0.19^b^

*Values represented are mean of 5 replicates in pot trial while 3 replicates in field trial; values followed by different alphabets in the rows are significantly different at P < 0.05 using Tukey's HSD*.

1 CI, Chlorophyll index;

2 PR, Photosynthesis rate;

3 SC, Stomatal conductance;

4 C_i_, Intercellular CO_2_ concentration;

5 ϕPS2, Quantum yield of PS II electron transport;

6 ϕCO_2_, Apparent quantum yield of CO_2_ assimilation;

7 qP, Photochemical quenching;

8 NPQ, Non-photochemical fluorescence quenching;

9 ETR, Photosynthetic electron transport rate;

10 TR, Transpiration rate;

11 WUE, Water use efficiency;

12 CLT, Computed leaf temperature;

13 C_i_/C_a_, Intercellular CO_2_ conc./Ambient CO_2_ conc.;

14 SOP, sulfate of potash;

15 SBD, Sea bittern-derived;

16 *PAS, Potassium ammonium sulfate*.

### Nutrient content and uptake

**Figure 1:**
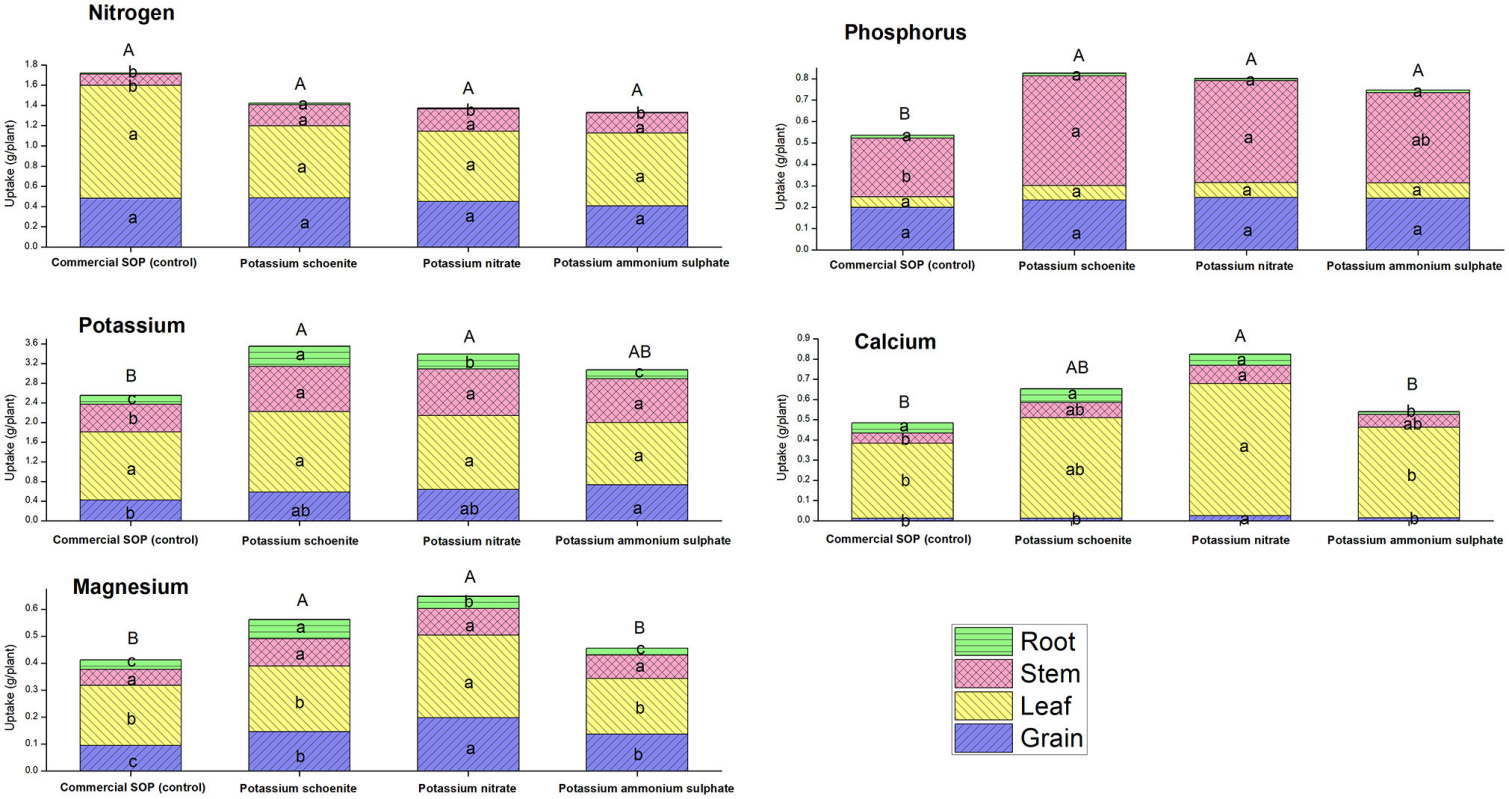
Nutrient uptake (g plant^−1^) of specific plant part *viz*. grain, leaf, stem and root of maize pot experiment as affected by different sea bittern-derived potassic nutrient treatments. Values are mean of 5 replicates. Bars followed by different alphabets within the treatment are significantly different at P < 0.05 using Tukey's HSD. Capital alphabets represents overall uptake of the particular nutrient in a treatment and small alphabets represents uptake of particular nutrient by the specific plant part viz. grain, leaf, stem and root.

**Figure 2:**
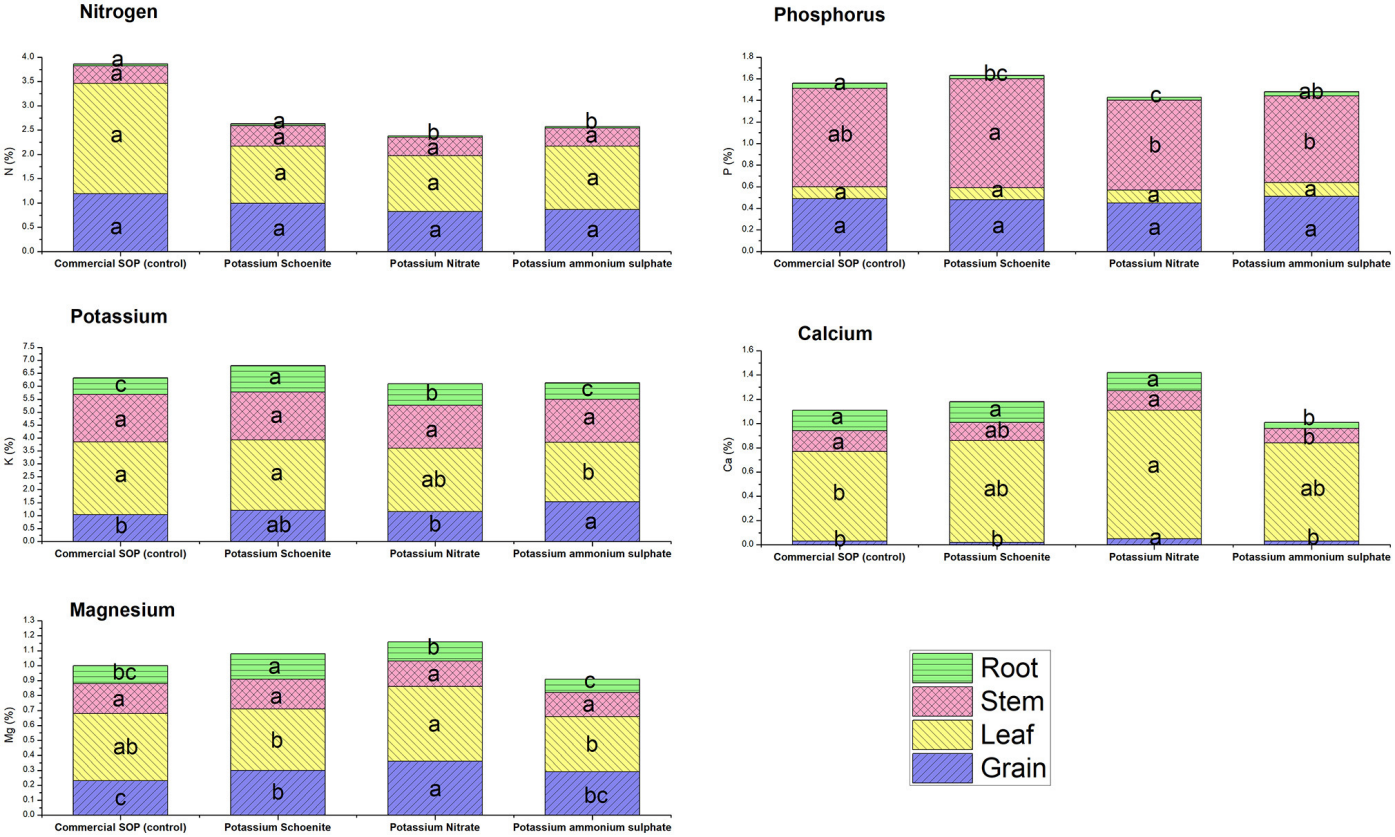
Nutrient content (%) of specific plant part *viz*. grain, leaf, stem and root of maize pot experiment as affected by different sea bittern-derived potassic nutrient treatments. Values are mean of 5 replicates. Bars followed by different alphabets within the treatment are significantly different at *P* < 0.05 using Tukey's HSD.

Nutrient content in the plant parts as well as their uptake in their respective plant parts were affected due to the different bittern-derived K treatments (Figures 1, 2). No specific pattern of changes were found in common for all these nutrients studied and it was apparent that different treatments brought out different levels of changes in different plant parts with respect to content and uptake. Total potassium uptake by plants was the highest in SBD-K schoenite treatment which was at par to other bittern-derived K compounds, all of which except SBD-PAS were statistically superior to control. Compared to control, K content in grains was found to be significantly higher in SBD-PAS treated plants, closely followed by SBD-K schoenite, -KNO_3_ and commercial SOP; however, these three treatments were at par to each other. Similar trend was also observed in the uptake of K in grains. The K content in leaf was found to be more or less similar in SBD-KNO_3_, -K schoenite and control, while SBD-PAS exhibited significantly lower leaf K content. Leaf K uptake however did not vary due to any of the treatments. Stem K content was at par in all the treatments, while the uptake was significantly higher in all the three bittern-derived potash treatments compared to that in control. Root K content was significantly higher in SBD-K schoenite treatment, followed by -KNO_3_, -PAS and control, the latter being at par to control. Similar trend was observed for the K uptake by roots. Among the different plant parts, N content varied only in roots, however, the differences were not significant enough to influence total N uptake by the plants due to various treatments. There was no change in P content and uptake in leaves and grains due to the various treatments. Significant changes were observed in content and uptake of P in stem and root. The significant difference in total P uptake in all the bittern-derived K treatments when compared to control was mainly due to differences in P uptake by stem. The total uptake of Ca and Mg was the highest in SBD-KNO_3_ treatments and the difference was significant compared to that in control. This treatment was however at par with SBD-K schoenite. The SOP and SBD-PAS were found statistically similar for total Ca and Mg uptake and were inferior to the other two treatments. Noticeably, the Ca and Mg content and uptake in grains were significantly higher in SBD-KNO_3_ treatment when compared to all other remaining treatments.

### Soil parameters

Effect of different potassic sources on soil properties has been shown in Table 5. Soil pH, moisture content (MC) and water holding capacity (WHC) were not affected by different treatments. Electrical conductivity was also not affected except in one treatment SBD-PAS, in which it decreased slightly as compared to commercial SOP. One of the explanations for the similar EC values in different treatments with differing quantities of applied fertilizers may be on account of soil buffering effect. Moreover, addition of K salts to the soil would not only result in a simple increase in the concentration of K, but might also modulate the concentrations of various other cations such as Ca, Mg, Na, Al, and H in the soil solution making it a complex phenomenon. The lowered EC in SBD- PAS may be as per the prevailing view, that if cationic nutrients as K and NH_4_ are added to soils, the bulk of them if not all, can be held as an exchangeable form by the negative charge of soils and hence would not be in the soil solution phase thus leading to lower EC values as opined by Yamasaki and Kishita (1972). Available K content of the soil measured after the harvest decreased in all the bittern-derived K treatments, which was significant in case of SBD-K Schoenite and SBD-PAS compared to control, however all the SBD-potassic forms were at par to each other. This might be due to higher uptake by the plants. Available nitrogen was significantly decreased in SBD-KNO_3_ and SBD-PAS sulfate treated soils, while available phosphorus was at par in all treatments. Soil organic carbon was also found at par among all the treatments. Soil enzymatic activity was also found to be significantly altered by different forms of bittern-derived potash treatments (Table 5). Activity of aryl sulphatase was significantly increased by all bittern-derived K treatments compared to SOP commercial, and all three of them were at par to each other. Similar trend was also recorded in acid phosphatase and FDA hydrolysis activity. The highest activity of alkaline phosphatase was recorded in SBD-KNO_3_ treated soil, followed by SBD-K schoenite, SBD-PAS and commercial SOP. Maximum glucosidase enzyme activity was found in SBD-KNO_3_ treatment, which was at par with SBD-K schoenite and significantly higher than SBD-PAS and control.

**Table 5:** Soil physico-chemical properties of maize as affected by different sea bittern-derived potassic nutrient treatments of pot experiment.

**Parameters**	**Commercial ^1^SOP (control)**	**^2^SBD-K schoenite**	**SBD-KNO_3_**	**SBD-^3^PAS**
^4^WHC at harvest (%)	48.20^a^	50.56^a^	54.96^a^	50.35^a^
^5^MC at harvest (%)	10.11^a^	10.41^a^	12.57^a^	11.65^a^
pH	8.88^a^	8.86^a^	8.87^a^	8.86^a^
^6^EC (dS m^−1^)	0.83^a^	0.80^ab^	0.79^ab^	0.74^b^
Available K (mg kg^−1^)	103.17^a^	74.81^b^	90.45^ab^	82.67^b^
Available P (mg kg^−1^)	3.75^a^	4.05^a^	3.92^a^	3.13^a^
Available N (mg kg^−1^)	72.38^a^	70.44^a^	58.26^b^	52.71^b^
^7^OC (%)	0.98^a^	1.03^a^	0.99^a^	0.99^a^
Aryl Sulphatase (μg nitrophenol released h^−1^ g^−1^ dry soil)	26.44^b^	42.81^a^	43.36^a^	41.01^a^
Alkaline Phosphatase (μg nitrophenol released h^−1^ g^−1^ dry soil)	291.85^b^	355.29^a^	385.32^a^	319.64^b^
Acid Phosphatase (μg nitrophenol released h^−1^ g^−1^ dry soil)	63.81^b^	87.24^a^	80.31^a^	77.57^a^
Glucosidase (μg nitrophenol released h^−1^ g^−1^ dry soil)	120.25^c^	211.22^a^	212.68^a^	153.77^b^
^8^FDA (μg fluorescence g^−1^ dry soil)	56.73^b^	64.97^a^	67.30^a^	63.55^a^

*Values represented are mean of 5 replicates. Values followed by different alphabets in the rows are significantly different at P < 0.05 using Tukey's HSD*.

1 SOP, Sulfate of potash;

2 SBD, Sea bittern-derived;

3 PAS, Potassium ammonium sulfate;

4 WHC, Water holding capacity;

5 MC, Moisture content;

6 EC, Electrical conductivity;

7 OC, Organic carbon

8 *FDA, Fluorescence diacetate*.

### Economic analysis

The highest net return per hectare was obtained in the SBD-KNO_3_ treatment (INR 33694), followed by SBD-K schoenite and SBD-PAS, with the benefit: cost ratio also following the same order. The commercial SOP had the lowest values for both these parameters. The incremental return realized over the control treatment per unit of incremental investment on account of K and the inadvertently adjusted N fertilizers was found the highest (3.8) in the schoenite treatment in spite of the lower enhancement in grain yield over commercial SOP, while SBD-KNO_3_ closely followed it recording a ratio of 3.7 (Table 6).

**Table 6:** Economic analysis of maize as affected by different sea bittern-derived potassic nutrient treatments in pot experiment.

**Parameters**	**Treatments**			
	**Commercial ^1^SOP (control)**	**^2^SBD-K schoenite**	**SBD-KNO_3_**	**SBD-^3^PAS**
General cost of cultivation (^4^INR)	39,288	39,288	39,288	39,288
Incremental invest (INR)	4,292	2,997	4,888	4,269
Total cost of cultivation (INR)	43,580	42,285	44,176	43,557
Grain yield (t ha^−1^)	3.64	4.25	4.80	4.31
Stone yield (t ha^−1^)	1.46	1.55	1.83	1.46
Stalk yield (t ha^−1^)	8.0	9.8	10.5	9.5
Total gross return (INR)	59,209	69,412	77,870	69,758
Net return (INR)	15,629	27,127	33,694	26,201
B : C ratio	1.36	1.64	1.76	1.60
Incremental return (INR)	0	11,499	18,065	10,573
Incremental return over control/incremental investment of N and K fertilizers	0.0	3.8	3.7	2.5

1 SOP, Sulfate of potash;

2 SBD, Sea bittern-derived;

3 PAS, Potassium ammonium sulfate;

4 *INR, Indian Rupee*.

## Discussion

The experimental results revealed that the sea-bittern derived potassic nutrient sources were superior to the conventionally used sulfate of potash in terms of growth, yield, gas exchange parameters and nutrient uptake. Further, the application of these K sources also improved the soil enzymatic activity. We hypothesize that the three SBD-fertilizers used might have improved growth and yield by different mechanisms. The SBD-PAS and SBD-schoenite was apparently found to be more balanced among the three SBD-fertilizers as it had the presence of all the three secondary nutrients, viz., Ca, Mg, and S in it in contrast to control and KNO_3._This might have enhanced various physiological parameters like photosynthetic rate, quantum yield of PS II electron transport, chlorophyll index (Table 4) which might have contributed to the growth and yield. On the other hand, the improvement in productivity brought out by the use of SBD-KNO_3_ might have been due to improved plant water status as evident from the measurements of water content in the plant parts and increase in root volume. This might be explained due to enhanced nitrate concentration around roots which induces alteration in root hydraulic properties which persists as long as nitrate levels are maintained (Gorska et al., 2008b). Further, they showed that the increased flux was linked to nitrate addition, but not to the addition of sulfate and phosphate anions or other forms of nitrogen which is commensurate with that observed in control treatment (Gorska et al., 2008a). It was confirmed that nitrate concentration within cells, rather than the products of nitrate assimilation, triggered the hydraulic response. There are evidences showing that nitrate can regulate transcript levels of some water channel (aquaporin) genes (Wang et al., 2001; Guo et al., 2007). In addition, higher root volume in SBD-KNO_3_ treated plants might have resulted in enhanced nutrient absorption area eventually leading to higher nutrient uptake. The enhanced hydration level together with the observed better stomatal conductance and consequent higher intercellular CO_2_ concentration led to least stressful leaf conditioning as evident by the highest Fv/Fm ratio. These might have led to better formation and assimilation of photosynthates that ultimately manifested into high dry matter accumulation by application of SBD-KNO_3_. Although leaf thickness was not carried out, we hypothesize that the leaf thickness might be higher for all the treatments compared to control. This emanates from the fact that although there was significant difference in dry leaf biomass plant^−1^ between control and other three treatments, there was no statistical difference in leaf area plant^−1^ among the treatments. This is also evident from specific leaf weight (data not shown) which was higher for all the treatments than control, wherein this value for SBD-KNO_3_ was 22.7% higher than that for control. This also probably manifested in having higher pigment concentration which was significantly higher in the three sea bittern-derived K treatments than control (Table 4). Although there was no statistical difference among all the treatments for cob diameter and number of seed rows per cob, the higher grain and carbohydrate yield obtained in SBD-KNO_3_ treated plants could be due to longer fill distance of seeds on the cob and bolder seeds as evidenced by the highest 100-seed weight compared to all other treatments. Heavier fresh cob with bigger seed size is important factor for getting premier price in market. Similarly, relatively higher yield obtained over control by application of SBD-PAS could be attributed to higher test weight, while higher yields by application of SBD-K schoenite was due to the highest number of seeds formed per cob and a modestly higher test weight. Similar results were obtained under field conditions as well, wherein all the bittern-derived potassic sources of nutrients were found significantly superior to traditional sulfate of potash. Slightly better response of SBD-K schoenite and SBD-PAS under field condition than under pot condition might be explained on account of higher pH in pot soils which might have made the ammonical or the urea form of N prone to greater volatilization losses.

The data on nutrient analysis revealed no significant change in nitrogen uptake among the various treatments while uptake of P, K and Mg was significantly higher over control in plants that received SBD-K schoenite and SBD-potassium nitrate. Interestingly, Ca and Mg uptake was significantly lower, though at par with control, under SBD-PAS treatments. This might have been due to the known negative impact of ammonium ions on the base cation uptake (Gloser and Gloser, 2000). This may be important in case the soil contains less than critical levels of these nutrients which would diminish the Mg uptake very adversely and thus application of SBD-PAS might not be suitable proposition for Ca and Mg deficient soils. This condition is especially possible in saline and sodic soils. Notably, NaCl content and thus Cl^−^ concentration in SBD- K schoenite was on a higher side, i.e., 3.66 and 3.52%, respectively, and therefore its continuous use over the long run might cause damage to plant growth, especially to the chloride sensitive crops. It may be noted that the Na and Cl^−^ content in SBD- K schoenite is dependent on the NaCl content in the feed composition of the kainite from which it is made.

The control treatment received N through urea which needs to be converted into ammonical form and then into nitrate form in which it is absorbed by maize plants. It is well known that this transformation is more prone to different kinds of volatilization and leaching losses resulting in lower fertilizer use efficiency. Compared to nitrate releaser SBD-KNO_3_, ammonium releaser SBD-PAS resulted in smaller root volume and lesser degree of hydration in plant. However, this disadvantage was partially offset by photon energy saving brought about by significantly higher net photosynthetic rate along with better water use efficiency also resulted in relatively higher grain yield compared to control. Similar results were also reported by Guo et al. (2007). Fertilizers effecting high water use efficiency yet giving modest yield, higher than usual, would certainly be instrumental for the crops grown in drought prone areas. Notably, K is also known to influence disease resistance and the enhanced availability and uptake of K by SBD K fertilizer treated plants might be other aspect that may be investigated in future.

Notably, there were no significant changes in most of the soil physico-chemical properties due to the application of bittern-derived potassic source of nutrients. There was slight decrease in electrical conductivity of the soil under SBD-PAS treatment. The highest available potash at crop harvest was found in the treatment receiving control which might be on the account of low potassium uptake from soil in this treatment. The lower available N in soil in SBD-KNO_3_ and SBD-PAS treated plants may be explained on account of being in more soluble forms resulting in greater exhaustion until harvest.

Activities of all the soil enzymes studied *viz*., aryl sulphatase, alkaline phosphatase, acid phosphatase, glucosidase and FDA which are involved in nutrient transformation cycle were significantly higher than that under control. Enzymes being the direct mediators for biological catabolism of soil organic and mineral fractions, allow a meaningful assessment of reaction rates for important soil processes and are closely related to microbial activity. Although it would require repeated application to assess the long term effect of these fertilizers on soil, nevertheless, soil enzymatic activity can reflect upon changes much sooner than other measurable parameters and therefore can give early indications of changes in soil health (Das and Varma, 2010). The favorable results of soil enzymatic activities obtained upon application of sea-bittern derived potassic nutrient sources unequivocally establishes the potential to use these compounds as fertilizer material to increase the productivity of field crops without harming soil health.

## Conclusion

It can be concluded that there was no deleterious effect on plant and soil by application of these bittern-derived potassic nutrient sources and that they can be unequivocally used as a fertilizer for raising crop productivity in a sustainable and economic manner. The sustainability is also on account of huge saving in terms of global warming potential for transporting potassic fertilizer through oceanic transport in countries that do not have potassic mineral reserves (Singh et al., 2015). These fertilizers are superior to the traditionally used sulfate of potash on being manufactured from non-traditional feedstock—sea bittern—which otherwise is a waste material. Also, they either contain nitrogen in more available forms (SBD-KNO_3_ and SBD-PAS) or contain additional nutrients like sulfur, magnesium and calcium (SBD-K schoenite and SBD-PAS) which gets inadvertently added to the soil when added as fertilizer, thereby eliciting better crop response due to being more balanced in its composition.

## Author contributions

KT performed experimental work, analyzed and interpreted data and wrote the manuscript. DK and KA helped in soil analysis. KA helped in data interpretation and critically reviewed the manuscript. RK and HT helped in the photosynthesis data and plant analysis. KG and PM prepared characterized and provided the fertilizers. AG designed the experiment, coordinated the work, interpreted data and wrote the manuscript.

### Conflict of interest statement

The authors declare that the research was conducted in the absence of any commercial or financial relationships that could be construed as a potential conflict of interest.

## Abbreviations

PAS: Potassium ammonium sulfate
SOP: Sulfate of potash
KNO_3_: Potassium nitrate
SSP: Single Super Phosphate
SBD: Sea bittern derived
SI: Salinity Index
FDA: Fluorescence diacetate
INR: Indian Rupee
CC: Cubic centimeters.
