# Supplementary material for: Evaluation of Fertilizer Potential of Different K Compounds Prepared Utilizing Sea Bittern as Feed Stock
Source: Front Plant Sci. 2017 Sep 7;8:1541. doi: 10.3389/fpls.2017.01541 (PMC5594215; doi:10.3389/fpls.2017.01541)
Supplement: Supplementary file 1 [file Table1.DOCX]

**Supplementary table 1:** Meteorological data of the field experimental area (November 2014 to February 2015).

| Week no. | Date  (DD/MM/YY) | Air Temperature (°C) | | | Precipitation  (mm) |
| --- | --- | --- | --- | --- | --- |
|  |  | Max | Min | Average |  |
| 1 | 01/11/14 to 07/11/14 | 37.8 | 22.6 | 29.4 | 0 |
| 2 | 8/11/14 to 14/11/14 | 36.6 | 22.0 | 29.3 | 0 |
| 3 | 15/11/14 to 21/11/14 | 35.9 | 21.0 | 29.1 | 0.00119 |
| 4 | 22/11/14 to 28/11/14 | 34.1 | 19.7 | 26.6 | 0 |
| 5 | 29/11/14 to 5/12/14 | 33.4 | 18.7 | 26.2 | 0 |
| 6 | 6/12/14 to 12/12/14 | 32.2 | 17.3 | 24.4 | 0 |
| 7 | 13/12/14 to 19/12/14 | 31.8 | 12.4 | 21.0 | 0 |
| 8 | 20/12/14 to 26/12/14 | 30.0 | 12.5 | 21.1 | 0 |
| 9 | 27/12/14 to 02/01/15 | 27.4 | 12.7 | 20.1 | 0 |
| 10 | 03/01/15 to 09/01/15 | 30.7 | 12.8 | 21.9 | 0 |
| 11 | 10/01/15 to 16/01/15 | 30.2 | 13.9 | 21.7 | 0 |
| 12 | 17/01/15 to 23/01/15 | 31.6 | 12.7 | 21.4 | 0.00390 |
| 13 | 24/01/15 to 30/01/15 | 28.7 | 12.3 | 20.1 | 0 |
| 14 | 31/01/15 to 06/02/15 | 32.8 | 13.0 | 23.5 | 0 |
| 15 | 07/02/15 to 13/02/15 | 33.3 | 15.6 | 24.2 | 0 |
| 16 | 14/02/15 to 20/02/15 | 37.3 | 18.3 | 26.9 | 0 |
| 17 | 21/02/15 to 28/02/15 | 38.6 | 17.5 | 26.9 | 0 |
